# Conventional and Nonconventional Therapies for COVID-19 Management in Trinidad

**DOI:** 10.1155/sci5/1545153

**Published:** 2024-11-21

**Authors:** Mohammad Sani Ismaila, Kavita Ranjeeta Lall, Kezia Sookram, Venkatesan Sundaram, Kegan Romelle Jones

**Affiliations:** Department of Basic Veterinary Sciences, School of Veterinary Medicine, Faculty of Medical Sciences, The University of the West Indies, St. Augustine Campus, St. Augustine, Trinidad and Tobago

**Keywords:** antibiotics, anti-inflammatory, Ivermectin, nonconventional treatment, social media

## Abstract

This cross-sectional study investigated nonconventional therapies for COVID-19 in Trinidad, emphasizing the need for documentation supporting future pharmaceutical research. The survey, conducted from June 20 to July 19, 2022, garnered responses from 57 participants aged 18 and above, with 82.46% vaccinated. The majority (81%) utilized both conventional and nonconventional therapies, revealing insights for potential alternatives to traditional treatments. Conventional treatments, including antibiotics, Ivermectin, anti-inflammatories, analgesics, bronchodilators, and cough/flu syrups, were frequently reported. Nonconventional therapies encompassed vitamins, minerals, supplements, and various plant and animal products. When participants used conventional therapies, either alone or in combination with nonconventional ones, 13.21% reported side effects. These included severe thirst, headache, nausea, drowsiness, and one case of weight gain. Conversely, those exclusively using nonconventional treatments reported no side effects. Encouragingly, nonconventional therapies demonstrated promising effects in managing COVID-19, emphasizing the need for meticulous selection, research, and development of their bioactive compounds as potential alternatives to conventional therapies.

## 1. Introduction

The COVID-19 infection stands as the deadliest pandemic in recent history, impacting the world profoundly. Despite the extensive efforts by scientists to develop various vaccines approximately 3 years postoutbreak, the disease continues to pose a threat in various regions globally. Although diverse vaccines have been created to provide immunity against the virus, instances of reinfection with the same or other variants and strains of the deadly organism persist [1, 2]. Studies indicate a heightened risk of reinfection among individuals previously infected or vaccinated compared to the unvaccinated [2–6]. Consequently, there has been a recognized necessity for booster doses at shorter intervals for those vaccinated, utilizing either the same or different types of vaccines [7–9].

The uncertainties surrounding reinfection and the prohibitive cost of conventional therapy for this infection, particularly in developing nations, have driven many individuals to explore nonconventional and alternative therapies for both treating and preventing COVID-19 [10–14]. Complementary and alternative medicine (CAM) or nonconventional therapies involve the utilization of substances or procedures in ailment management that lack authentication or recommendation by medical authorities, often employed by patients without prescription or monitoring [15, 16].

The use of CAM is gaining popularity in managing challenging ailments such as cancer and viral diseases [17–19]. Since the onset of the COVID-19 pandemic, various alternative and nonconventional therapies have been employed globally to manage the infection [11, 14, 20]. In Trinidad, despite the prevalent use of nonconventional products for managing and preventing COVID-19, there is currently no documented information on these products, including their activity or potential toxicity. Therefore, this study aims to survey the conventional and nonconventional therapies used in Trinidad, with the goal of classifying them for a more in-depth analysis of their activity, toxicity, and potential side effects. This research seeks to fill a crucial gap in understanding and managing COVID-19 in Trinidad, providing valuable insights into a significant aspect of the global response to the pandemic.

## 2. Materials and Methods

Ethical approval for this study was granted by the Campus Research Ethics Committee, U.W.I., with the reference number CREC-SA 1650/06/2022. In accordance with ethical standards, stringent measures were implemented to ensure participant confidentiality and informed consent throughout the survey process.

To gather comprehensive insights, a cross-sectional online survey utilizing Google Forms was conducted across Trinidad and Tobago. The survey targeted individuals aged 18 years and above, leveraging popular social media channels, including Facebook, WhatsApp, and Instagram. The choice of online surveys and social media platforms was made considering their wide accessibility; however, it is important to note that this approach may introduce biases related to digital literacy and internet access.

The survey spanned from June 20th to July 19th, 2022, allowing for a thorough examination of nonconventional therapies employed during this period. Data collected through the survey underwent analysis using Microsoft Excel, chosen for its practicality and wide availability. In detailing the results, mean values and standard errors of the mean (SEM) were calculated and compared across the various parameters measured. This statistical approach enhances the robustness of the analysis, providing a nuanced understanding of the variations and trends within the dataset.

It is important to acknowledge the limitations associated with online surveys and the potential biases introduced by the reliance on social media channels. While the chosen methods allow for a broad sampling, caution should be exercised in generalizing the findings to the entire population.

This methodology was meticulously designed to capture a holistic view of nonconventional therapies in Trinidad, aiming to facilitate a comprehensive classification and analysis of their activity, toxicity, and potential side effects.

## 3. Results

**Figure 1:**
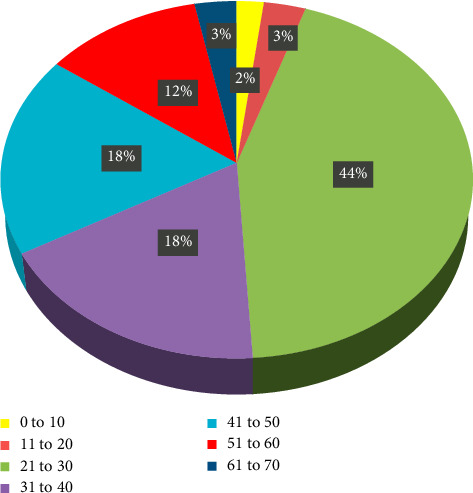
Age demographics of COVID-19 diagnoses in Trinidad, 2022.

**Figure 2:**
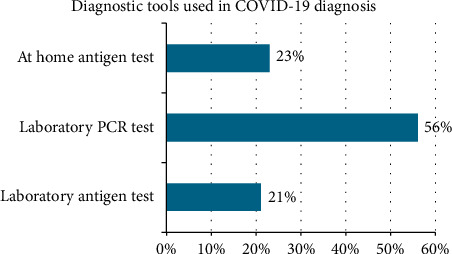
Diagnostic tools used in COVID-19 diagnosis.

This study cantered on individuals aged 18 and above in Trinidad, offering an in-depth exploration of nonconventional therapy utilization in the local context. The age distribution of COVID-19 diagnoses ranged from 0 to 70 years old, as visualized in Figure 1, while the diagnostic methods employed are detailed in Figure 2.

A total of 57 respondents participated, with 17.54% being unvaccinated and 82.46% vaccinated. The distribution of vaccine types is outlined in Table 1.

**Figure 3:**
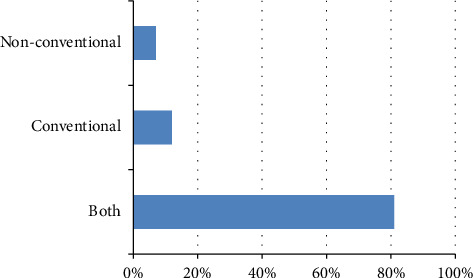
Method of therapy used for COVID-19.

The treatment landscape for COVID-19 involved diverse approaches, as illustrated in Figure 3. Conventional therapies, detailed in Table 2, included orally administered pharmaceutical drugs, while nonconventional therapies, outlined in Table 3, embraced herbal teas, essential oils, and vitamins. Notably, 81% of respondents integrated both conventional and nonconventional therapies, 7% relied solely on nonconventional treatments, and 12.28% used conventional therapies exclusively.

This study unveiled pivotal factors guiding the selection of nonconventional therapies among respondents in Trinidad. Tradition, deeply rooted personal beliefs, accounts of successful recovery, and a desire to fortify the immune system emerged as influential determinants in therapy choices. Instructions on therapy application were diverse, ranging from familial and social circles to information gleaned from social media platforms. Daily usage of these nonconventional therapies demonstrated a noteworthy variability, spanning from one to five times.

In examining conventional therapies, either alone or in conjunction with nonconventional counterparts, 13.21% of individuals reported side effects, including severe thirst, headache, nausea, drowsiness, and one case of weight gain. Conversely, those exclusively utilizing nonconventional treatments reported no side effects. This dichotomy in side effect profiles necessitates a thorough investigation into the safety and tolerability of both therapeutic approaches.

A reassuring finding is that all participants who tested positive for COVID-19, regardless of their chosen treatment method, achieved successful recovery. While this outcome is encouraging, it is imperative to acknowledge potential confounding factors that may have influenced recovery, such as variations in symptom severity, comorbidities, or individual immune responses.

These results illuminate the multifaceted landscape of COVID-19 management in Trinidad, underscoring the complex interplay of cultural, individual, and therapeutic factors.

## 4. Discussion

Previous reports have highlighted the utilization of alternative therapy in managing COVID-19. The findings of this study contribute to this growing body of knowledge, specifically examining the experiences of individuals who contracted the virus and the diverse array of therapies employed. Notably, the prevalence of middle-aged individuals (21–30 years old) contracting the virus aligns with earlier reports in South Korea [21].

The conventional therapies documented in our research, including antibiotics (Azithromycin, Levofloxacin, Augmentin), anti-inflammatory/analgesics (Prednisolone, Paracetamol, Zerodol), and antiparasitic (Ivermectin), resonated with earlier reports in the literature concerning the management of COVID-19 [22].

In contrast, the nonconventional therapies adopted by Trinidadians comprised a diverse spectrum of vitamins, minerals, and bioactive compounds from plants and animals. Despite established physiological roles, recent clinical trials have yielded mixed results. For instance, high-dose vitamin D3 did not significantly reduce hospitalization time in patients with COVID-19, challenging previous assumptions [23–26]. Intravenous administration of vitamin C, however, showed promise by reducing inflammatory markers, including ferritin and D-dimer. Vitamin B, known for its immune-boosting properties, also demonstrated positive effects on respiratory function and reduced hospital stay [27].

The use of various plant bioactive compounds by Trinidadian respondents, such as ginger, lemongrass, garlic, citrus, and eucalyptus oil, aligns with global studies on nonconventional therapies for COVID-19 [11, 28, 29]. Notably, many of these compounds are commonly used in local households for various ailments, with their application in COVID-19 treatment supported by existing literature [30, 31]. Additionally, familiar nonconventional therapies like Vicks, steaming, and lozenges were reported, consistent with findings from other studies [32, 33]. The inclusion of CBD oil, known for its therapeutic effects, prompts discussion on its opportunities, challenges, and efficacy in treating COVID-19, emphasizing the need for further research [34–36].

Honey, recognized for its bioactive compounds, emerged as a noteworthy nonconventional therapy in our study. Its combination with lime aligns with previous reports on the use of honey for managing infections [28, 37].

However, as we interpret these findings, it is crucial to acknowledge several nuances and potential limitations. While our study sheds light on the diverse therapeutic approaches adopted by individuals in Trinidad, the efficacy, safety, and standardization of these nonconventional therapies require rigorous investigation. Furthermore, the variations in individual responses and potential biases in self-reported data must be considered.

The multifaceted landscape of COVID-19 management in Trinidad, as illuminated by our results, underscores the complex interplay of cultural, individual, and therapeutic factors. These findings bear relevance not only for the local population but also contribute to the broader discourse on healthcare decision-making during pandemics. As we navigate the complexities revealed by our study, further research, including clinical trials and epidemiological investigations, is imperative to validate the safety and efficacy of these nonconventional therapies in the context of COVID-19. This collective effort will ultimately inform evidence-based healthcare practices and enhance our understanding of alternative therapeutic approaches in the ongoing battle against the pandemic.

## 5. Conclusions

This study unveils a spectrum of nonconventional therapies for COVID-19 in Trinidad, notably identifying indigenous plants specific to the region. While offering a diverse array of potential treatments, the conclusion resonates with a call for intensified research. Further characterization, exploration of mechanisms of action, and toxicity studies are urged to unlock the unique therapeutic properties of these local plants in the context of COVID-19 management.

## Figures and Tables

**Table 1:** Vaccine types and percentages.

Vaccine type	Total number vaccinated	Total percentage vaccinated (%)
AstraZeneca	17	36.17
Pfizer	4	8.51
Sinopharm	24	51.06
Sinopharm with Pfizer boosters	2	4.26

**Table 2:** Conventional drugs used in COVID-19 management.

Class of drug	Conventional therapies used
Antibiotics	Levofloxacin, Augmentin, Azithromycin Zithrocin

Anti-inflammatory/analgesics	Prednisone, Prednisolone, Advil (adult and children), Paracetamol (Panadol, Panadol multisymptom, Panadol extra-strength), Dexamethasone, Panadeine, aleve, Nimulid (NSAID), Zerodol (Paracetamol and Aceclofenac)

Antiparasitics	Ivermectin

Bronchodilators/cough syrup	Ventolin (salbutamol), Aleve, Solvin (cough syrup), Bisolvon (mucolytic), Afrin (nasal decongestant)
	DayQuil (dextromethorphan/acetaminophen), NyQuil sleep aid (diphenhydramine), Bronchosolvin (Guaifenesin expectorant), Histatussin DM (dextromethorphan), Covonia (cough syrup), codeine cough syrup, Mucinex (Guaifenesin/dextromethorphan), Tuscosed Linctus, Breathezy (Montelukast)

Anticoagulants	XARELTO (rivaroxaban)

**Table 3:** Nonconventional therapies used for COVID-19 management.

Class of bioactive	Nonconventional therapies used
Vitamins	Vitamin C, multivitamins, vitamin B complex, vitamin D

Minerals	Zinc, magnesium

Plants	Herbal teas (e.g., ginger *Zingiber officinale*), lemongrass (*Cymbopogon citratus*), garlic (*Allium sativum*), fever grass, turmeric (*Curcuma longa*), clove (*Syzygium aromaticum*), caraille (*Momordica charantia*), fresh juices (e.g., orange *Citrus sinensis*) lime (*Citrus aurantiifolia*), bandania roots (*Eryngium foetidum*)
	Spanish thyme (*Coleus amboinicus*), liquid chlorophyll, CBD oil *(Cannabis sativa*), papaya leaf (*Carica papaya*), *Echinacea* (*Echinacea purpurea*)
	*Eucalyptus* oil (*Eucalyptus camaldulensis*), vervine (*Verbena officinalis*), Zebapique (*Neurolaena lobate*)

Animals	Honey

Others	Elderberry, Vicks vapour rub, Menthol crystals, steaming, humidifier, peppermint oil, saline nasal rinse, Fisherman's friends lozenges for sore throat

## Data Availability

All data were presented in the manuscript.
