# Corrigendum to “Conventional and Nonconventional Therapies for COVID-19 Management in Trinidad”

**DOI:** 10.1155/sci5/9861363

**Published:** 2025-06-26

M. S. Ismaila, K. R. Lall, K. Sookram, V. Sundaram, and K. R. Jones, “Conventional and Nonconventional Therapies for COVID-19 Management in Trinidad,” *Scientifica* 2024, no. 1 (2024): 1–6, https://doi.org/10.1155/sci5/1545153.

In the article titled “Conventional and Nonconventional Therapies for COVID-19 Management in Trinidad,” there was a spelling error in the author list: Mohammad Sani Ismaila should have been listed as Muhammad Sani Ismaila. This has been corrected as shown below.

Muhammad Sani Ismaila, Kavita Ranjeeta Lall, Kezia Sookram, Venkatesan Sundaram, and Kegan Romelle Jones.

We apologize for this error.

